# Analysis of United Network for Organ Sharing: Berlin Heart as Bridge to Transplant in Patients Aged 5 and Under

**DOI:** 10.1016/j.atssr.2025.08.009

**Published:** 2025-08-30

**Authors:** Zachary Brennan, Ayah A. Ibrahim, Omar M. Sharaf, John Treffalls, Qiudong Chen, Dominic Emerson, Richard Kim, Giles J. Peek, Mark S. Bleiweis, Jeffrey P. Jacobs

**Affiliations:** 1 Departments of Surgery and Pediatrics, Congenital Heart Center, University of Florida, Gainesville, Florida; 2 Department of Cardiac Surgery, Smidt Heart Institute, Cedars-Sinai Medical Center, Los Angeles, California; 3 Burrell College of Osteopathic Medicine, Las Cruces, New Mexico; 4 Department of Surgery, Mayo Clinic, Rochester, Minnesota

## Abstract

**Background:**

The Berlin Heart paracorporeal ventricular assist device (VAD) is widely used for bridging pediatric patients to transplantation. Contemporary, multiinstitutional analyses of VAD types used in children and their impact on outcomes are limited. This analysis evaluates multiinstitutional outcomes after cardiac transplantation in patients aged ≤5 years in the following categories: no VAD, Berlin Heart VAD, or other VAD.

**Methods:**

The United Network for Organ Sharing database was analyzed to evaluate perioperative outcomes and 3-year survival in patients aged ≤5 years undergoing first-time cardiac transplantation (September 6, 2004 to June 30, 2023). The study population comprised 3649 patients (no VAD [n = 2822], Berlin Heart [n = 670], other VAD [n = 157]).

**Results:**

Distinct demographic and clinical characteristics were observed among patients bridged with Berlin Heart, including a higher prevalence of congenital heart disease, longer waitlist durations, and higher utilization of extracorporeal membrane oxygenation at transplant. Patients bridged with other VADs exhibited notably higher 3-year mortality compared with both Berlin Heart and no VAD groups. Multivariable analysis identified key risk factors associated with increased mortality, including congenital heart disease and extracorporeal membrane oxygenation at transplant.

**Conclusions:**

By highlighting the comparative effectiveness of different VAD types and key risk factors, our study contributes valuable insights in guiding clinical decision-making in this challenging patient population and also provides important information to guide future research.

**In Short**

▪ The Berlin Heart is a safe and effective bridge-to-transplant option for children aged ≤5 years, with favorable posttransplant survival.
▪ Patients supported with other ventricular assist devices (VADs) had notably lower 3-year post-transplant survival than both Berlin Heart and no-VAD groups.
▪ Key risk factors include congenital heart disease, extracorporeal membrane oxygenation at transplant, pretransplant organ dysfunction, and prolonged ischemic time.

The Berlin Heart EXCOR—a pulsatile paracorporeal ventricular assist device (VAD)—is the most commonly used pediatric VAD.^1^^,^^2^ Since the first patient was bridged to heart transplant with a Berlin Heart in 2004, its use has increased in both patients who eventually undergo transplantation and those who do not.^3^ Overall, VAD utilization has increased in recent years, and studies have shown no increased mortality in pediatric patients bridged to-transplant with a VAD in comparison to those undergoing cardiac transplantation without a VAD.^4^, ^5^, ^6^, ^7^, ^8^

Individual institutional studies have evaluated the Berlin Heart as a bridge to transplant, but the most recent United Network for Organ Sharing (UNOS) analysis was published with data ending in 2014 and only included 358 patients.^9^ Single-center studies have described complications, including hemorrhagic events, strokes, infections, and other end-organ dysfunction when bridging patients with the Berlin Heart and other VADs.^5^^,^^7^

While risk factors for pediatric heart transplantation and VAD support are established, an analysis of younger patients bridged with the Berlin Heart using UNOS data is lacking, and no study has compared outcomes between no VAD, Berlin Heart, or other VAD support in this challenging cohort. Understanding the Berlin Heart’s efficacy relative to other VADs and risks associated with heart transplant waiting periods aids clinicians in devising optimal bridging strategies and management techniques. The purpose of this analysis was to use UNOS to evaluate multiinstitutional outcomes after cardiac transplantation in patients aged ≤5 years in the following categories: no VAD, Berlin Heart VAD, or other VAD.

## Patients and Methods

### Study Population

A query of the UNOS database identified first-time heart transplant patients in the United States, aged 5 years or younger, between September 6, 2004, and June 30, 2023. Patients over 5 years, those without survival data, and those undergoing retransplantation were excluded. The final cohort included 3649 patients in 3 groups: no VAD (n = 2822; 77.3%), Berlin Heart (n = 670; 18.4%), and other VADs (n = 157; 4.3%). Other VADs included Jarvik 2000, Medos, Cardiac Assist Tandem Heart, Thoratec, Toyobo, Heartware HVAD, CentriMag, Maquet Jostra Rotaflow, PediMag, and Heartmate III. Institutional review board approval was not required, as all data were publicly available and deidentified through UNOS.

### Statistical Analysis

Continuous variables are reported as mean ± SD. Categorical variables are reported as numbers and percentages. Group differences were analyzed using χ^2^ or analysis of variance, with significance at *P* < .05. A univariable Cox proportional hazards model analyzed each variable independently as the predictor of survival. Variables with *P* < .20 and clinically relevant baseline and demographic characteristics were included in the multivariable Cox model, following recipients until death or censorship on June 30, 2023. Kaplan-Meier survival analysis assessed survival differences between patients with no VAD, Berlin Heart, or other VAD, with “time-zero” as the cardiac transplantation date. Posttransplant survival was evaluated at 3 years, with group differences tested via a log-rank test. Laboratory values (creatinine, albumin, bilirubin) reflect the most recent values prior to transplant. These variables were analyzed as continuous predictors, with no predefined thresholds. All statistical analyses were conducted using R (r-project.org), and the Kaplan-Meier survival graph was created in SPSS (IBM).

## Results

### Baseline and Demographic Characteristics

**Figure 1:**
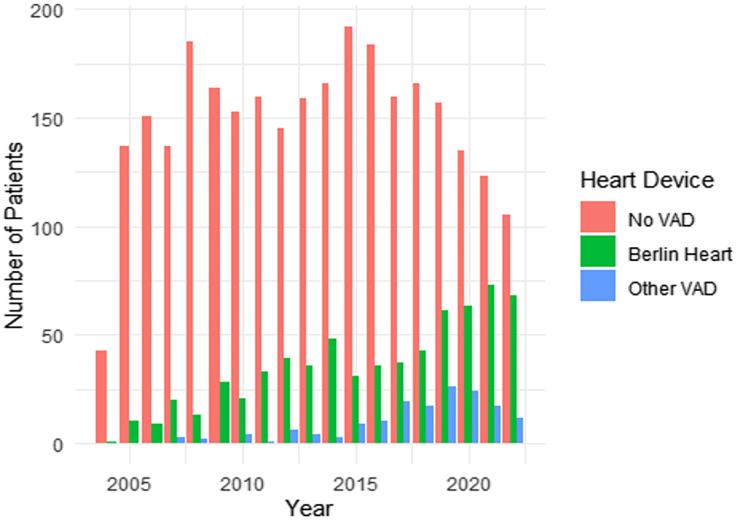
Patients bridged to transplant per year by device type. (VAD, ventricular assist device.)

Table 1 shows baseline and demographic characteristic differences by device type. Patients with no VAD (0.88 years) were younger (1.08 years with Berlin Heart vs 1.27 years with other VADs, *P <.*001) and more frequently White, non-Hispanic (55.8% no VAD; 46.6% Berlin Heart; 47.8% other VAD; *P < .*001). Sex varied across groups, with male patients composing 52.9% (no VAD), 49.3% (Berlin Heart), and 59.9% (other VADs); *P < .*001. Body mass index showed no significant difference (*P* = .884). The prevalence of congenital heart disease (CHD) was significantly higher in patients bridged with the Berlin Heart (73.3%; *P < .*001) compared with other VADs (49.7%) or no VAD (35.5%). Figure 1 shows the number of patients bridged annually by device type, highlighting an increasing trend in Berlin Heart usage since 2020, while no VAD and other VADs numbers varied annually.

**Table 1:** Baseline/Demographic Characteristics and Operative Details by Device Type

Variable	No VAD (n = 2822)	Berlin Heart (n = 670)	Other VAD (n = 157)	*P* Value
**Baseline/Demographic Characteristics**				
Age, y	0.88 ± 1.41	1.08 ± 1.45	1.27 ± 1.69	<.001
Male	1494 (52.9)	330 (49.3)	94 (59.9)	.039
Ethnic category				.001
White, non-Hispanic	1576 (55.8)	312 (46.6)	75 (47.8)	
Black	501 (17.8)	134 (20.0)	39 (24.8)	
Hispanic	570 (20.2)	161 (24.0)	29 (18.5)	
Asian	86 (3.0)	36 (5.4)	10 (6.4)	
American Indian/Alaska Native	19 (0.7)	5 (0.7)	2 (1.3)	
Pacific Islander	10 (0.4)	3 (0.4)	0 (0.0)	
Multiracial	60 (2.1)	19 (2.8)	2 (1.3)	
Weigh, kg	7.55 ± 4.41	8.72 ± 4.28	9.28 ± 5.83	<.001
Body mass index	15.51 (23.15)	15.15 (2.33)	15.92 (6.63)	.884
Noncongenital diagnosis, %	1819 (64.5)	179 (26.7)	79 (50.3)	<.001
Congenital heart disease	1003 (35.5)	491 (73.3)	78 (49.7)	<.001
**Operative Details**				
Days in Status 1A	55.15 ± 62.47	97.09 ± 75.68	76.86 ± 67.44	<.001
Total waitlist days	92.07 ± 126.46	113.46 ± 100.90	89.82 ± 75.78	<.001
Creatinine, mg/dL	0.38 ± 0.24	0.40 ± 1.04	0.36 ± 0.21	.522
Albumin, g/dL	3.47 ± 0.72	3.50 ± 0.82	3.37 ± 0.71	.133
ECMO at transplant	56 (1.89)	50 (7.46)	9 (5.73)	<.001

Values are displayed as mean ± SD or number (%).

ECMO, extracorporeal membrane oxygenation; VAD, ventricular assist device.

### Operative Details

Table 1 also shows operative characteristic differences by device type. Patients with a Berlin Heart spent more days in status 1A (97.1 ± 75.7 days; *P < .*001) compared to those with no VAD (55.2 ± 62.5 days), or other VADs (76.9 ± 67.4 days), had a longer total waitlist duration (113.5 ± 100.9 days Berlin Heart; 92.1 ± 126.5 days No VAD; 89.8 ± 75.8 days other VAD; *P < .*001) and were more likely to be on extracorporeal membrane oxygenation (ECMO) preoperatively prior to transplant (7.5% Berlin Heart; 1.9% no VAD; 5.7% other VAD; *P < .*001). Preoperative creatinine and albumin were similar between groups.

### Univariable and Multivariable Survival Analysis

Table 2 reports hazard ratios (HRs) from the univariable analysis, with each variable as the sole predictor of survival. In univariable analysis, increased posttransplant mortality at 3 years was associated with CHD diagnosis (HR, 2.371; 95% CI, 2.273-2.47; *P < .*001), other VAD (non-Berlin Heart) use (HR, 1.657; 95% CI, 1.478-1.835; *P* = .005), higher creatinine (HR, 1.095; 95% CI, 1.054-1.135; *P* = .024) and bilirubin (HR, 1.049; 95% CI, 1.043-1.055; *P < .*001), ECMO at transplant (HR, 2.855; 95% CI, 2.729-2.982; *P < .*001), pretransplant transfusions (HR, 1.686; 95% CI, 1.59-1.781; *P < .*001), pretransplant ventilator support (HR, 2.048; 95% CI, 1.958-2.137; *P < .*001), pretransplant inhaled nitric oxide use (HR, 3.967; 95% CI, 3.72-4.124; *P* = .001), dialysis prior to transplant (HR, 3.625; 95% CI, 3.452-3.798; *P < .*001), and increased donor heart ischemic time in hours (HR, 1.138; 95% CI, 1.103-1.174; *P < .*001). Meanwhile, in univariable analysis, decreased posttransplant mortality at 3 years was associated with pre-transplant Berlin Heart support (HR, 0.796; 95% CI, 0.671-0.92; *P* = .046), higher albumin (HR, 0.802; 95% CI, 0.741-0.863; *P < .*001), and older age per year (HR, 0.886; 95% CI, 0.854-0.917; *P < .*001).

**Table 2:** Univariable and Multivariable Analysis

Variable	Hazard Ratio (95% CI)	*P* Value
**Univariable Analysis**		
Berlin Heart	0.796 (0.671-0.92)	.046
Other VAD	1.657 (1.478-1.835)	.005
CHD	2.371 (2.273-2.47)	<.001
Higher creatinine	1.095 (1.054-1.135)	.024
Higher albumin	0.802 (0.741-0.863)	<.001
Higher total bilirubin	1.049 (1.043-1.055)	<.001
ECMO at transplant	2.855 (2.729-2.982)	<.001
Pretransplant transfusions	1.686 (1.59-1.781)	<.001
Pretransplant ventilator	2.048 (1.958-2.137)	<.001
Pretransplant inhaled NO	3.967 (3.72-4.214)	<.001
Older age	0.886 (0.854-0.917)	<.001
Dialysis pretransplant	3.625 (3.452-3.798)	<.001
Increased ischemic time	1.138 (1.103-1.174)	<.001
**Multivariable Analysis**		
Berlin Heart	0.935 (0.768-1.431)	.764
Other VAD	1.729 (1.161-2.575)	.007
CHD	2.089 (1.652-2.642)	<.001
Higher total bilirubin (per 1 mg/dL)	1.041 (1.023-1.059)	<.001
ECMO at transplant	1.932 (1.360-2.749)	<.001
Pretransplant transfusions	1.141 (0.894-1.547)	.289
Pretransplant ventilator	1.137 (0.956-1.471)	.326
Pretransplant inhaled NO	2.149 (1.206-3.823)	.009
Pretransplant dialysis	2.185 (1.469-3.249)	<.001
Increased ischemic time (by hour)	1.183 (1.054-1.328)	.004

CHD, congenital heart disease; ECMO, extracorporeal membrane oxygenation; NO, Nitric oxide; VAD, ventricular assist device.

Table 2 also displays the multivariable Cox proportional hazards regression. In multivariable analysis, increased posttransplant mortality at 3 years was associated with CHD diagnosis (HR, 2.089; 95% CI, 1.652-2.642; *P < .*001), other VAD use (HR, 1.729; 95% CI, 1.161-2.575; *P = .*007), higher bilirubin (HR, 1.041; 95% CI, 1.023-1.0059; *P < .*001), ECMO at transplant (HR, 1.932; 95% CI, 1.360-2.749; *P < .*001), pretransplant inhaled nitric oxide (HR, 2.149; 95% CI, 1.206-3.823; *P = .*009), pretransplant dialysis (HR, 2.185; 95% CI, 1.469-3.249; *P < .*001), and increased ischemic time (HR, 1.183 95% CI, 1.054-1.328; *P < .*001). Meanwhile, in multivariable analysis, Berlin Heart usage, transfusions, and ventilator support pretransplant showed no significant HR.

### Kaplan-Meier Analysis

**Figure 2:**
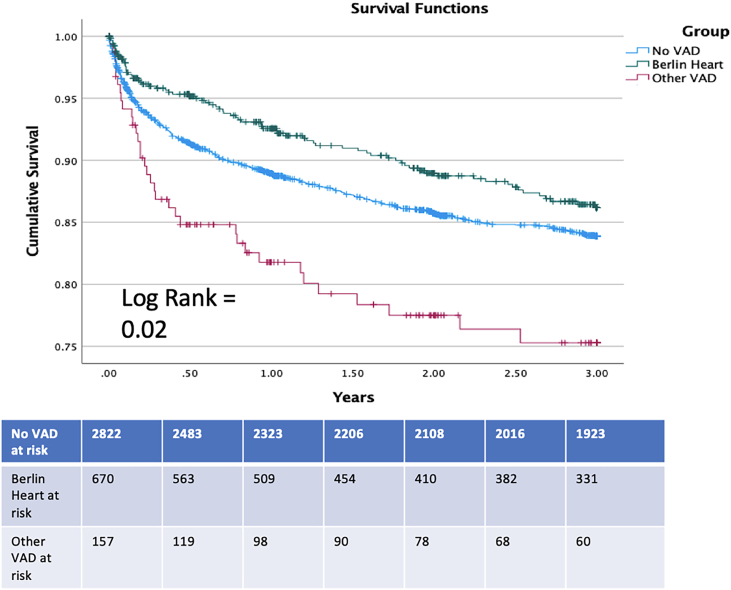
Three-year Kaplan Meier posttransplant survival analysis of each group. (VAD, ventricular assist device.)

Figure 2 illustrates the estimated 3-year survival curve per group, with notable survival differences (log-rank = 0.02). Survival at 3 years posttransplant was 86.2% (Berlin Heart), 83.9% (no VAD), and 75.3% (other VADs).

## Comment

Patients aged ≤5 years undergoing cardiac transplantation present unique challenges, particularly when supported with pretransplantation VAD. The utilization of VADs has increased to mitigate waitlist mortality, with the Berlin Heart as the most common choice in children.^4^ Younger patients with lower body weights are particularly challenging, with unique requirements, risk factors, and increased mortality.^1^^,^^5^, ^6^, ^7^ These patients typically present with poorer health at baseline and have distinct risk factors vs their non-VAD counterparts.^4^^,^^8^^,^^10^

Our study provides an important addition to the literature as a contemporary multiinstitutional analysis of the Berlin Heart as a bridge to transplant in patients ≤5 years, assessing its comparative efficacy vs other VADs and no VAD, which previous studies have lacked. In our analysis of 3649 first-time heart transplant patients aged ≤5 years in the UNOS database, we observed patients with a Berlin Heart were less likely to be White, non-Hispanic, more likely to have CHD, on ECMO at transplant, and spend more days in status 1A and on the waitlist overall. Three-year unadjusted survival was highest with the Berlin Heart (86.2%) and lowest with other VAD (75.3%). A previous study showed similar survival rates for the Berlin Heart compared to no VAD but did not compare it to other VAD types.^9^ Our analysis demonstrates the important finding in patients ≤5 years that other VADs have increased mortality risk overall and worse survival than both Berlin Heart and no VAD.

Our multivariable analysis showed several other important risk factors for 3-year posttransplant mortality. Notably, CHD, ECMO at transplant, pretransplant inhaled nitric oxide, pretransplant dialysis, and increased ischemic time were associated with an increased risk of mortality. Therefore, clinicians must recognize these risk factors when evaluating patients for transplantation, as mitigating them (when possible) can improve patient outcomes. Patient selection is also critical, both for listing cardiac transplantation candidates and determining VAD support, particularly in patients with nonmodifiable risk factors such as CHD.

This analysis carries the inherent limitations of any large retrospective administrative database review. Our study relied on the UNOS database, where data accuracy and missingness varied among patients and variables. The absence of implant and explant dates prevented assessment of support duration—a key risk factor. Additionally, the lack of data on devices removed before transplant, including intraaortic balloon pumps and ECMO, hinders insight into device usage between waitlisting and transplantation, as only devices active at those specific times were recorded. Similarly, detailed subanalysis of “other VAD” patients is limited due to the small number bridged with specific types of other VADs. The small number of patients supported by other VADs limits robust analysis for this category.

In conclusion, patients aged ≤5 years can be safely bridged to transplant with a Berlin Heart. Other VAD types had a higher mortality risk in these younger patients. The risk factors and underlying characteristics outlined in this study can guide clinician decision-making regarding patients requiring cardiac transplantation in this age group. Additionally, the overall trends can inform further research into specific bridging strategies for these challenging patients.
